# Supplementary figures and images for: Relaxing restrictions at the pace of vaccination increases freedom and guards against further COVID-19 waves
Source: PLoS Comput Biol. 2021 Sep 2;17(9):e1009288. doi: 10.1371/journal.pcbi.1009288 (PMC8412259; doi:10.1371/journal.pcbi.1009288)

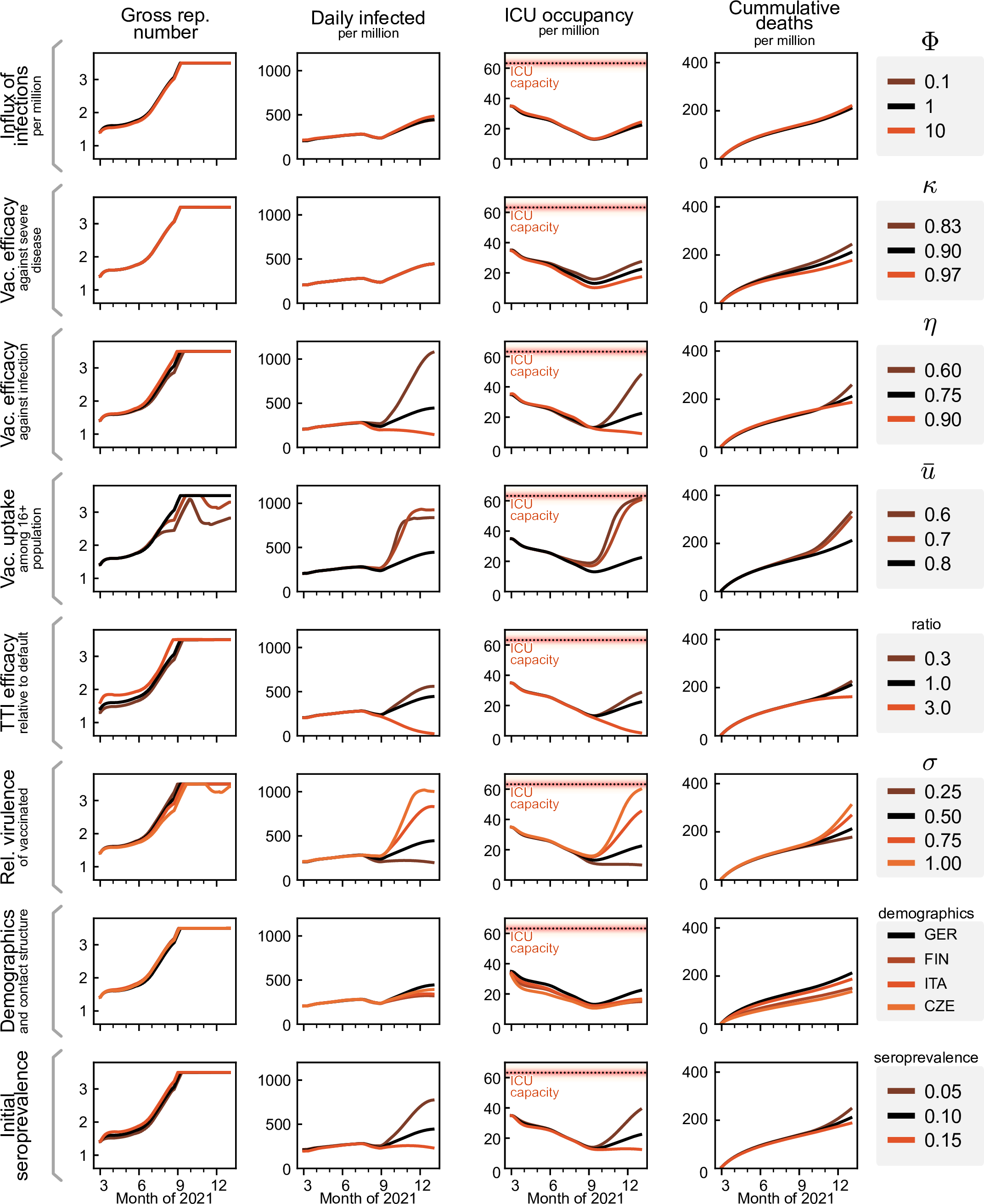

Supplement: S1 Fig — We vary central parameters of the model individually, while keeping all others at their respective default value. For assessing the sensitivity to the TTI efficacy we scale all the capacity limits NTTI, Ntest(eff), Ntest(ineff) and Nno test (see Methods) by a common ratio. (TIF) [file pcbi.1009288.s001.tif]

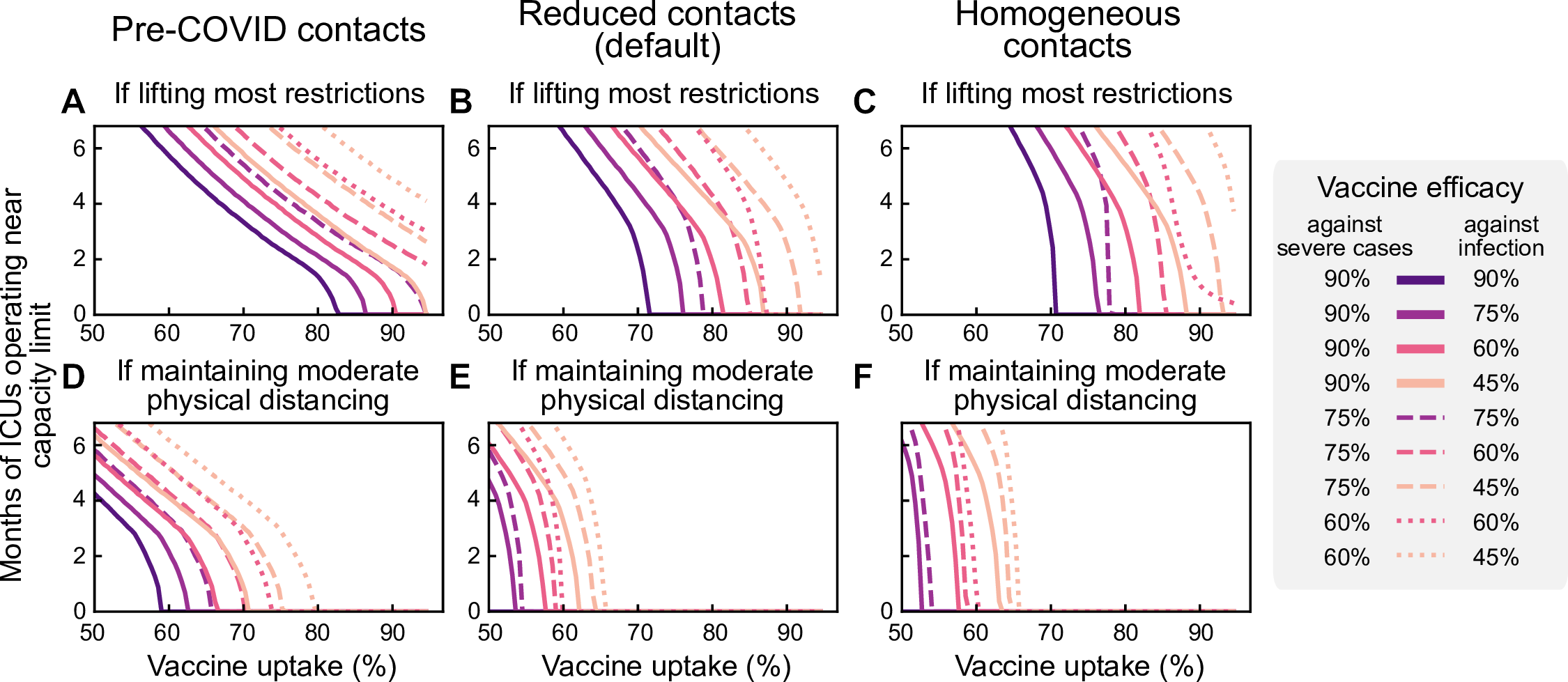

Supplement: S2 Fig — We assume that infections are kept stable at 250 daily infections until all age groups have been vaccinated. Then most restrictions are lifted, leading to a wave if vaccine uptake has not been high enough (see Fig 4A). We measure the severity of the wave (quantified by the duration of full ICUs) for varying uptake and vaccine efficacies for different contact structures (see Fig 7A–7C). A-C: The duration of the wave (measured by the duration of full ICUs) depends on the vaccine uptake and on the effectiveness of the vaccine measured by its efficacy at preventing infection (shades of purple) and severe illness (vaccine efficacy, full vs dashed vs dotted). D-F: If some NPIs are kept in place (such that the gross reproduction number goes up to Rt = 2.5), ICUs would be prevented from overflowing even in some cases of lower vaccine effectiveness. If precautionary measures are dropped in all age groups, including schools (A,D) the required uptake to prevent a further severe wave is increased by about 10% when compared to our default scenario of some continued measures to reduce the potential contagious contacts in school settings (B,E) or to completely homogeneous contacts (C,F). Not all combinations of vaccine effectiveness are possible as the vaccine efficacy against severe illness is by definition larger as the protection against any infection at all. (TIF) [file pcbi.1009288.s002.tif]

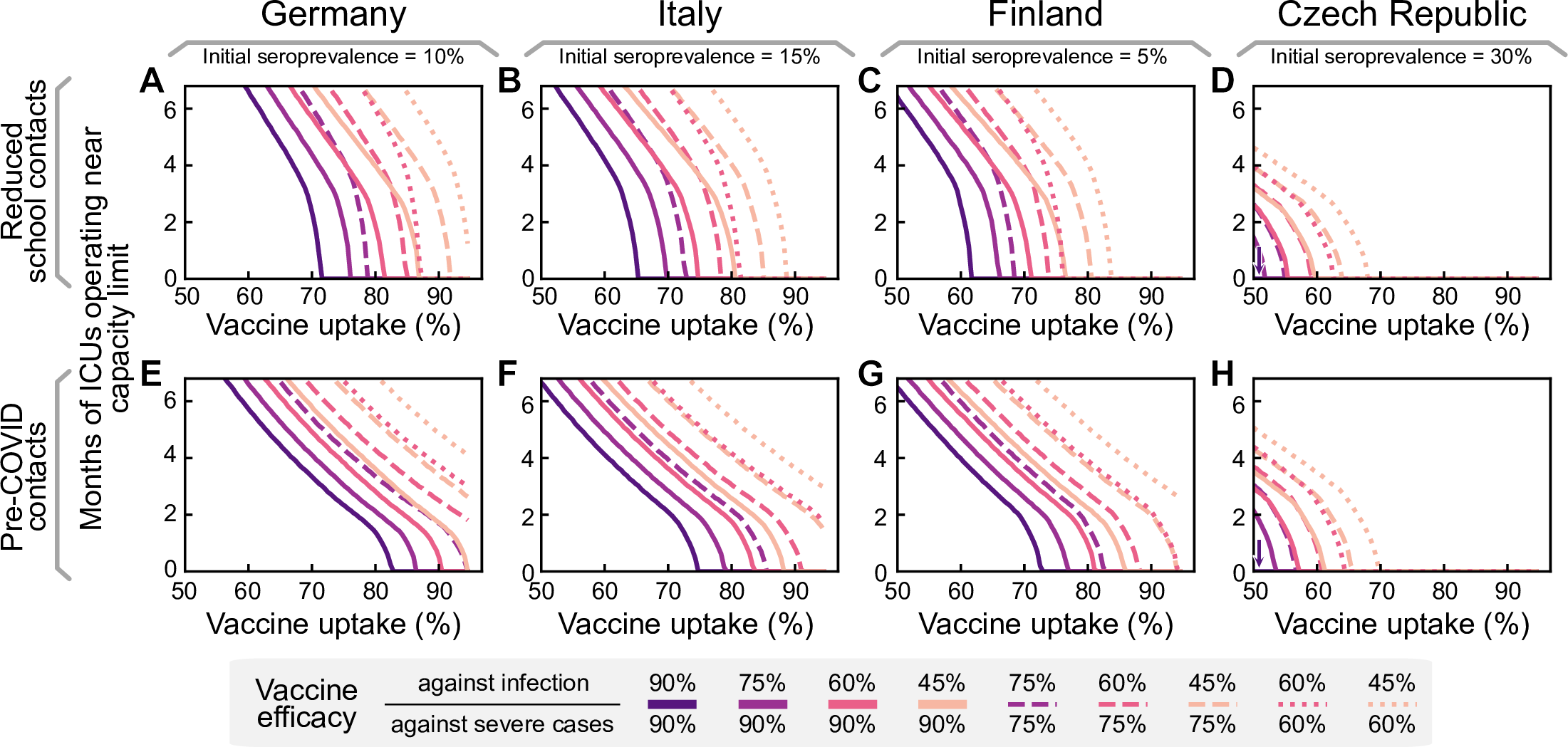

Supplement: S3 Fig — Extended version of Fig 5, including more combinations of vaccine efficacies. A–D: If releasing all measures to pre-COVID contacts, keeping only some measures aiming to cup the reproduction number at 3.5. E–H: If releasing all measures to pre-COVID contacts, keeping only some measures aiming to cup the reproduction number at 3.5 and halving the contagiousness of contacts at school ages. (TIF) [file pcbi.1009288.s003.tif]

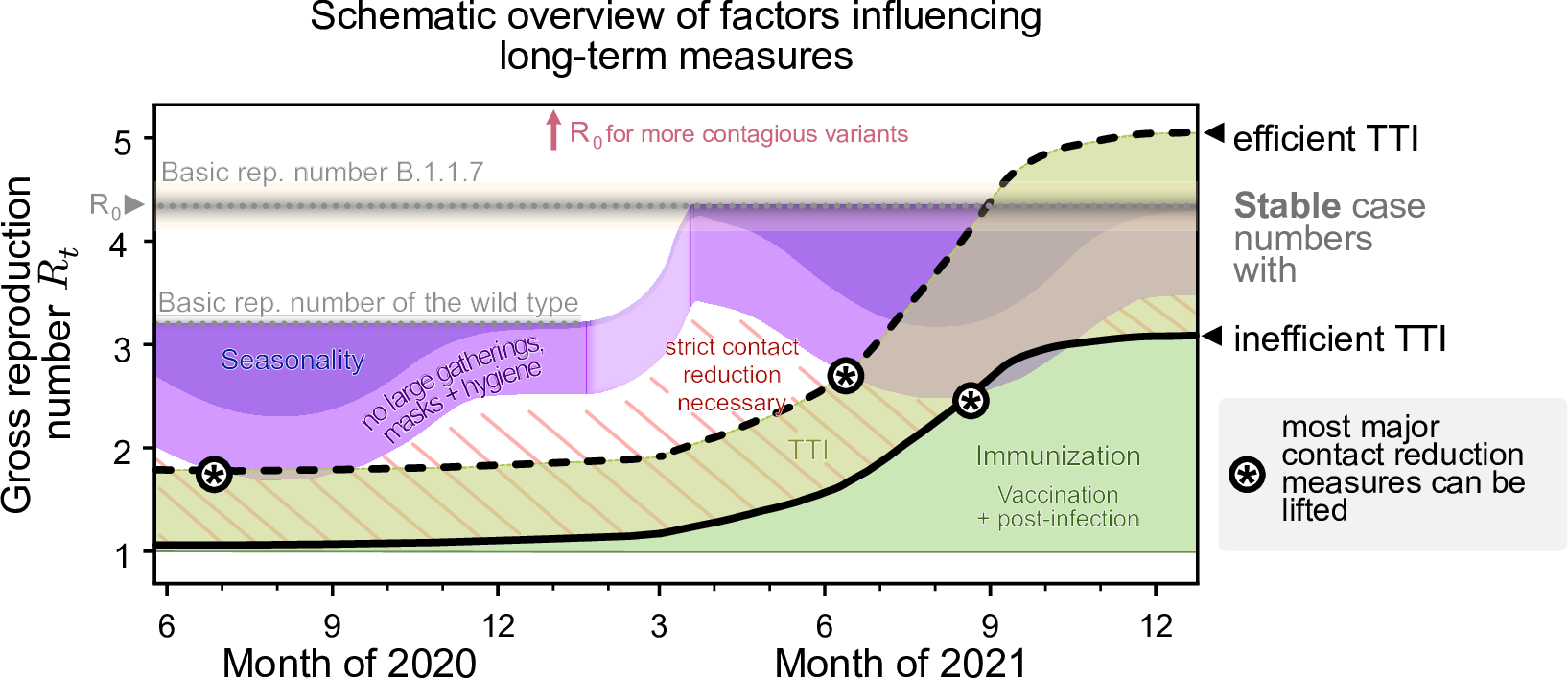

Supplement: S4 Fig — Schematic outlook into the effects of vaccination and the B.1.1.7 variant of SARS-CoV-2 on the societal freedom in the EU in 2021 compared to 2020 (see also the caption for Fig 1A). In 2020, seasonality effects and efficient test-trace-and-isolate (TTI) programs at low case numbers allowed for stable case numbers with only mild restrictions during summer, until about September. In 2021, vaccinations are expected to allow for greater freedom, but also a more contagious variant (B.1.1.7) is prevalent across the EU. Efficient TTI at low case numbers would thus help lifting major restrictions earlier. The exact transition period between the wild type and B.1.1.7 (light purple shaded area) varies regionally. (TIF) [file pcbi.1009288.s004.tif]

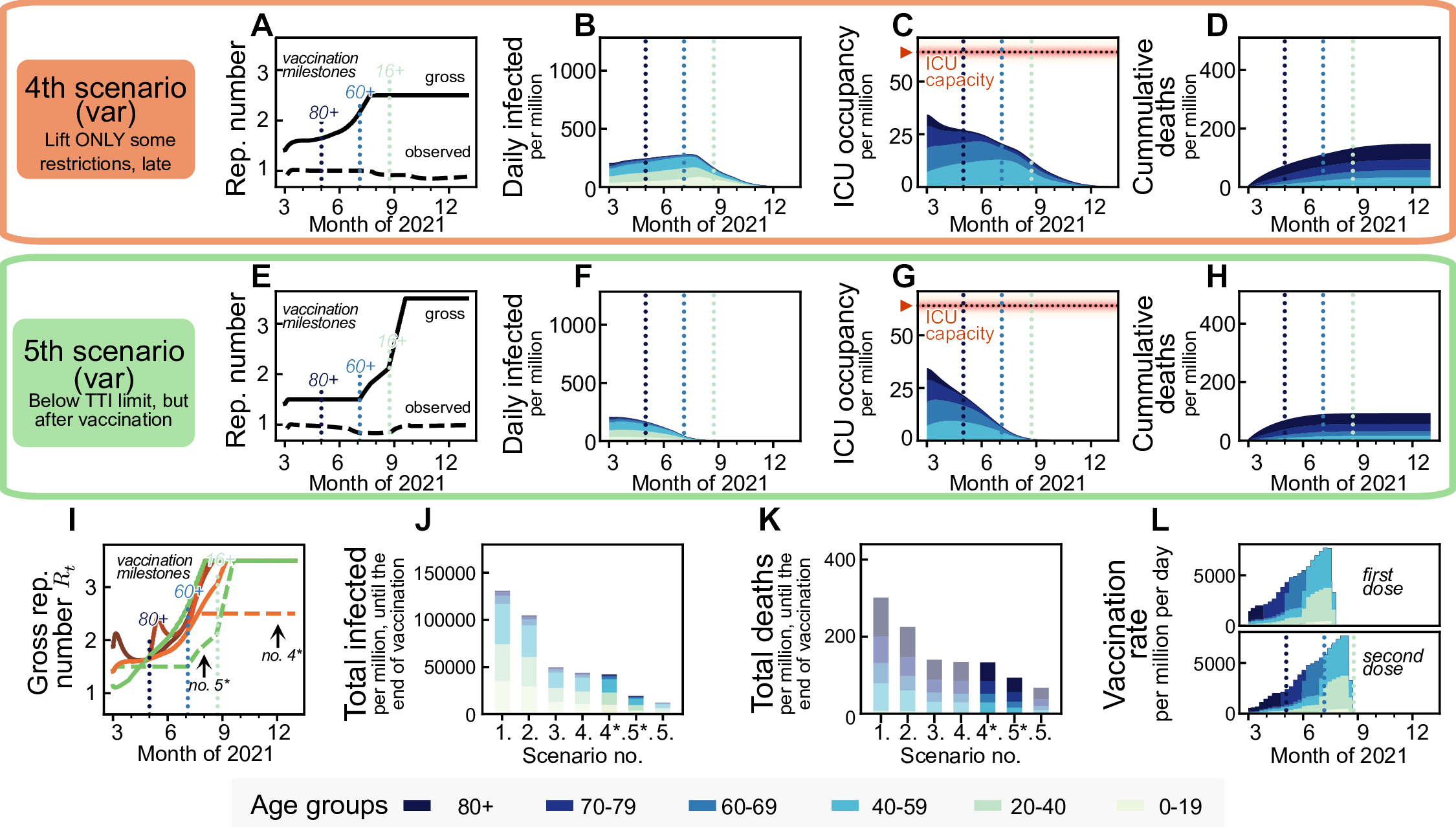

Supplement: S5 Fig — A–D: Variation of the fourth scenario from the main text (see Fig 3), where moderate restrictions are kept in place in the long term (letting the gross reproduction number go up to 2.5, compared to 3.5 in the default scenarios). E–H: Variation of the fifth scenario from the main text (see Fig 2) avoiding the strict initial restrictions. Keeping the gross reproduction number at a moderate level (1.5) until the everyone above 60 has been offered vaccination allows to decrease case numbers steadily. Over the summer a slight gradual increase in the contacts is allowed and all NPIs expect for test-trace-and-isolate (TTI) and enhanced hygiene are lifted when everyone received the vaccination offer (increasing the gross reproduction number to 3.5). I: The variation of the fourth scenario initially allows for the same increase in freedom as all the main scenarios, but needs more restrictions in the long term. The variation of the fifth scenario calls for stricter NPIs in the mid-term, but grants high freedom after summer. J,K: Both proposals lead to low number of infections and fatalities. L: Projected vaccination rates (see Fig 2). (TIF) [file pcbi.1009288.s005.tif]

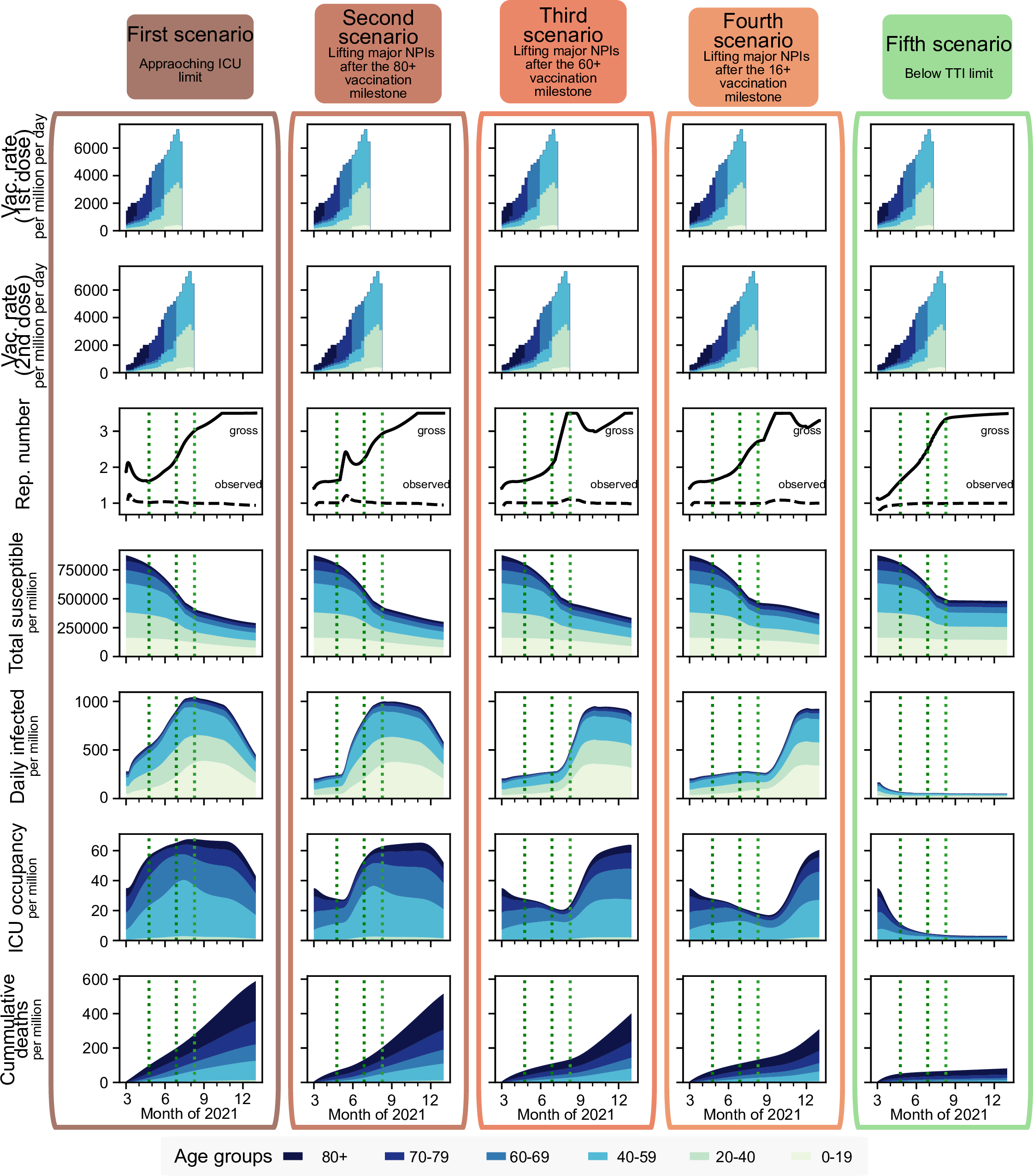

Supplement: S6 Fig — Scenarios using default protection against infection η = 0.75 and low vaccine uptake of 70% among the adult population. (TIF) [file pcbi.1009288.s006.tif]

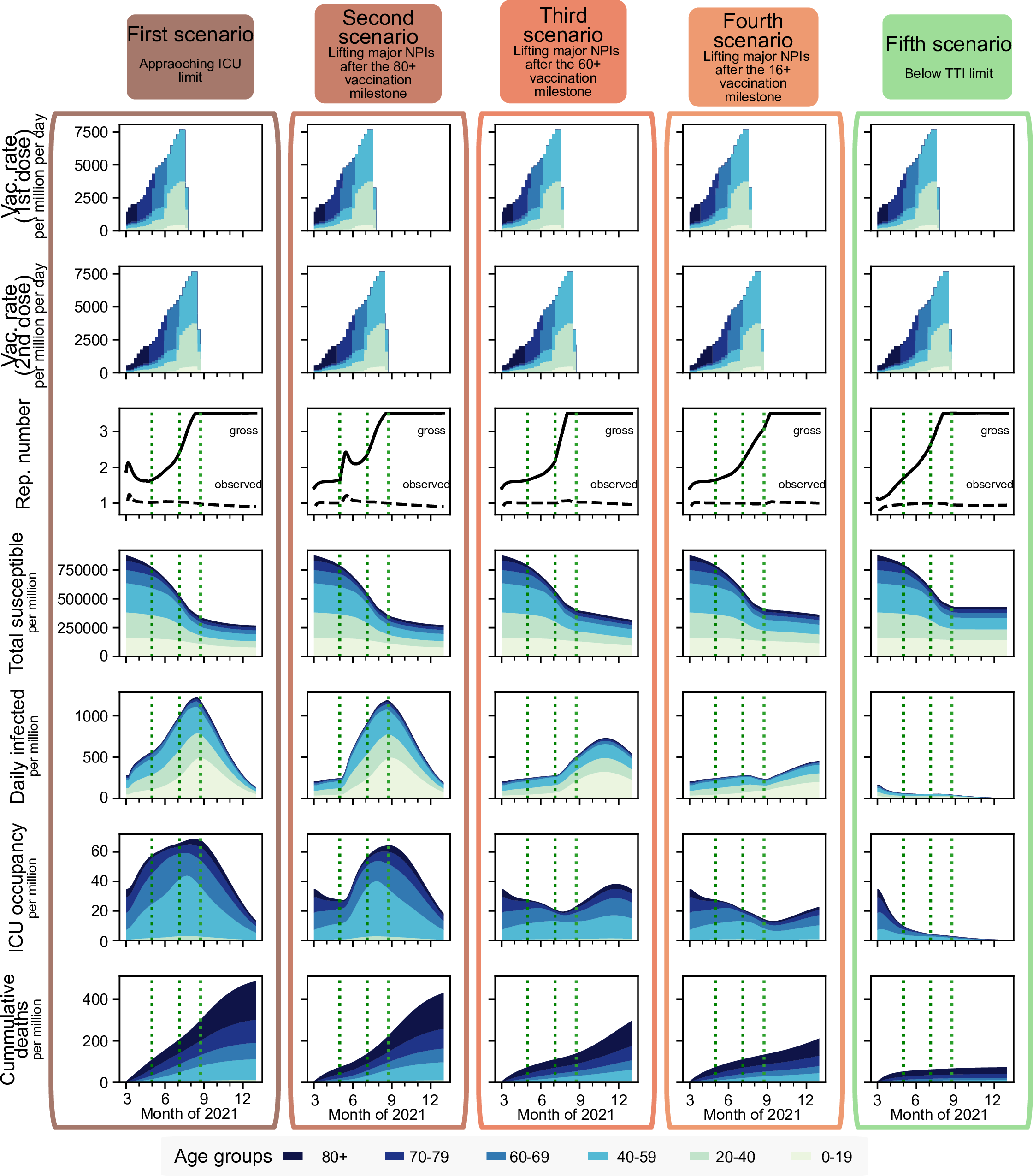

Supplement: S7 Fig — Scenarios using default protection against infection η = 0.75 and default vaccine uptake of 80% among the adult population. (TIF) [file pcbi.1009288.s007.tif]

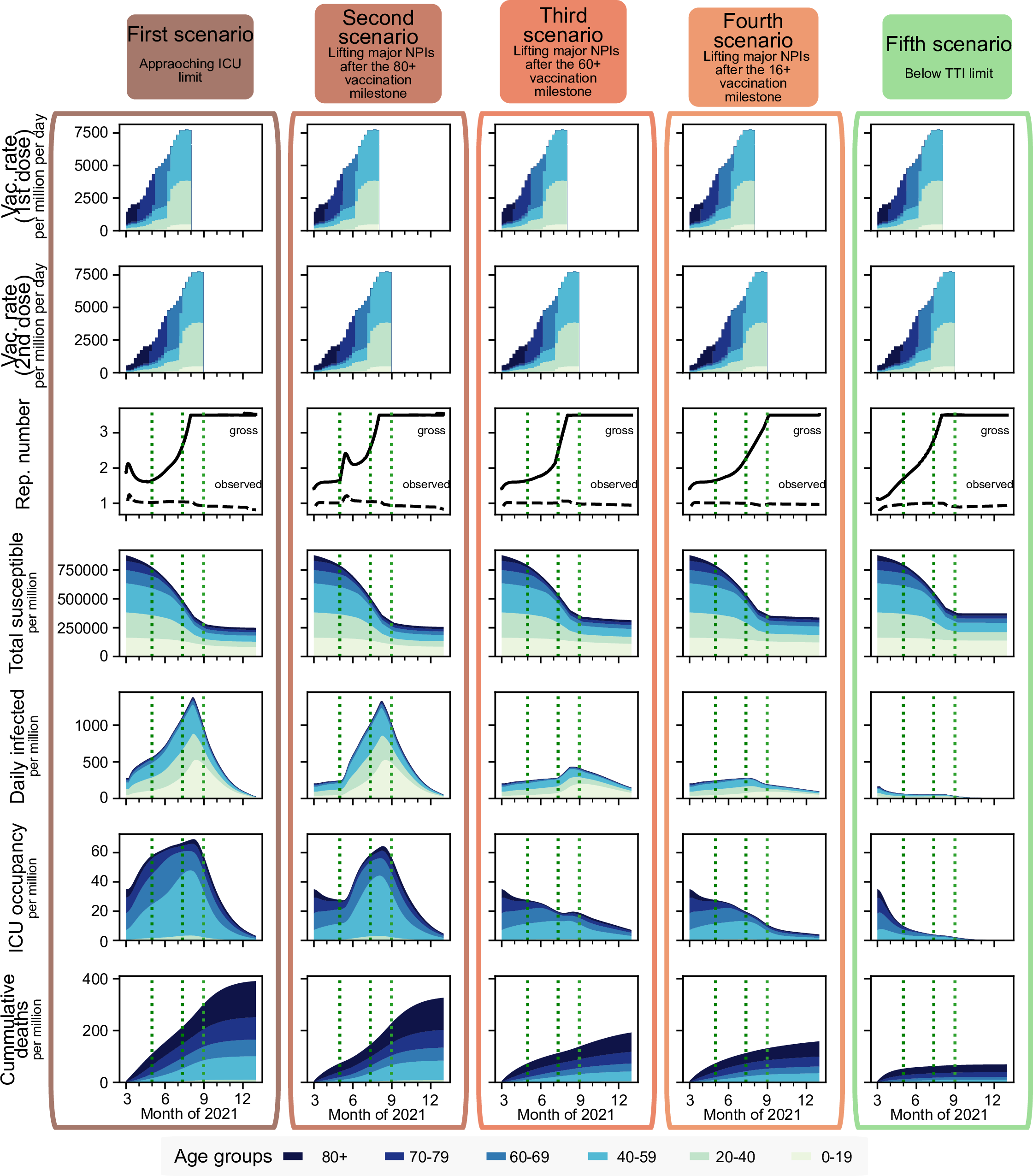

Supplement: S8 Fig — Scenarios using default protection against infection η = 0.75 and high vaccine uptake of 90% among the adult population. (TIF) [file pcbi.1009288.s008.tif]

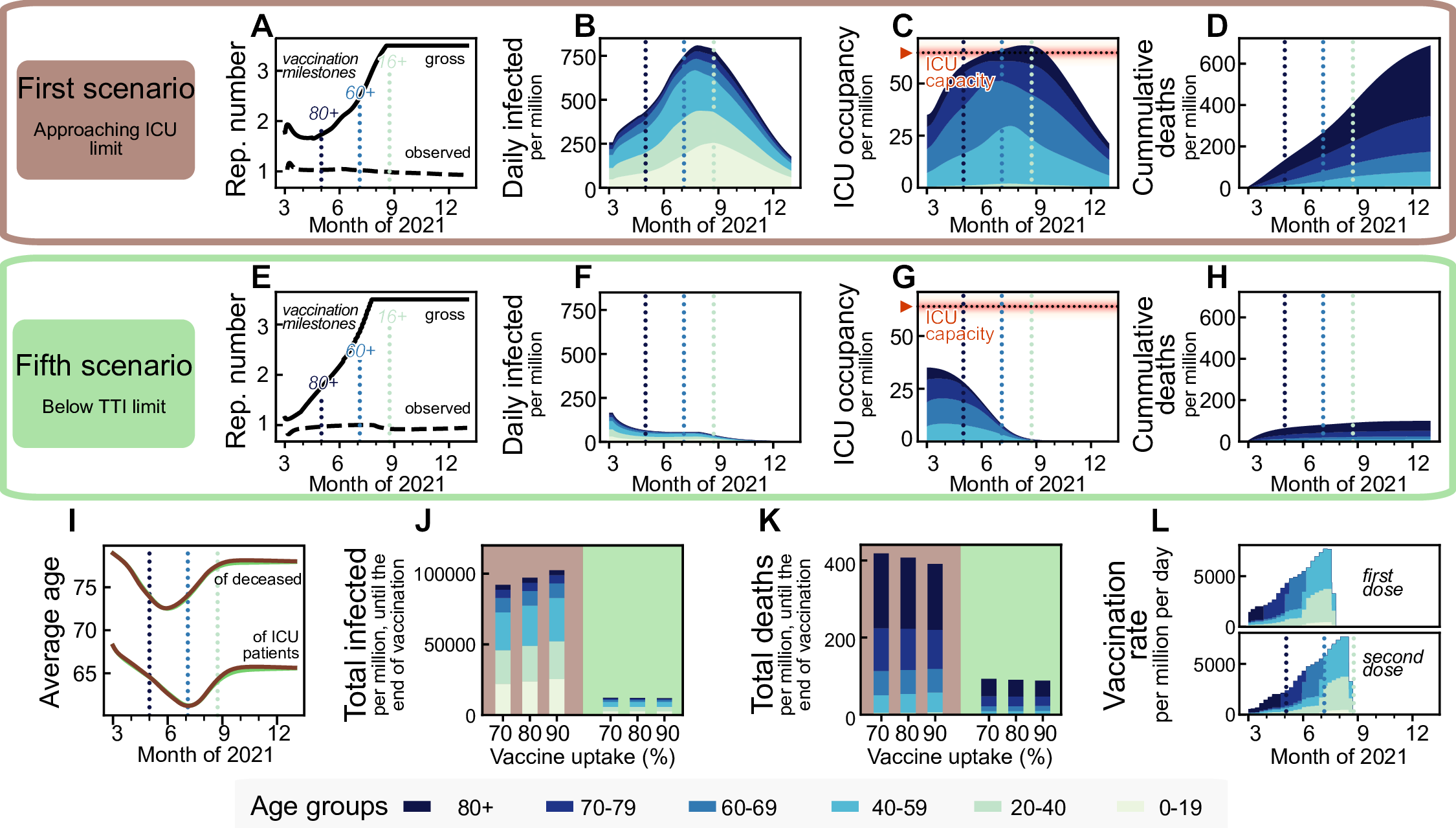

Supplement: S9 Fig — (TIF) [file pcbi.1009288.s009.tif]

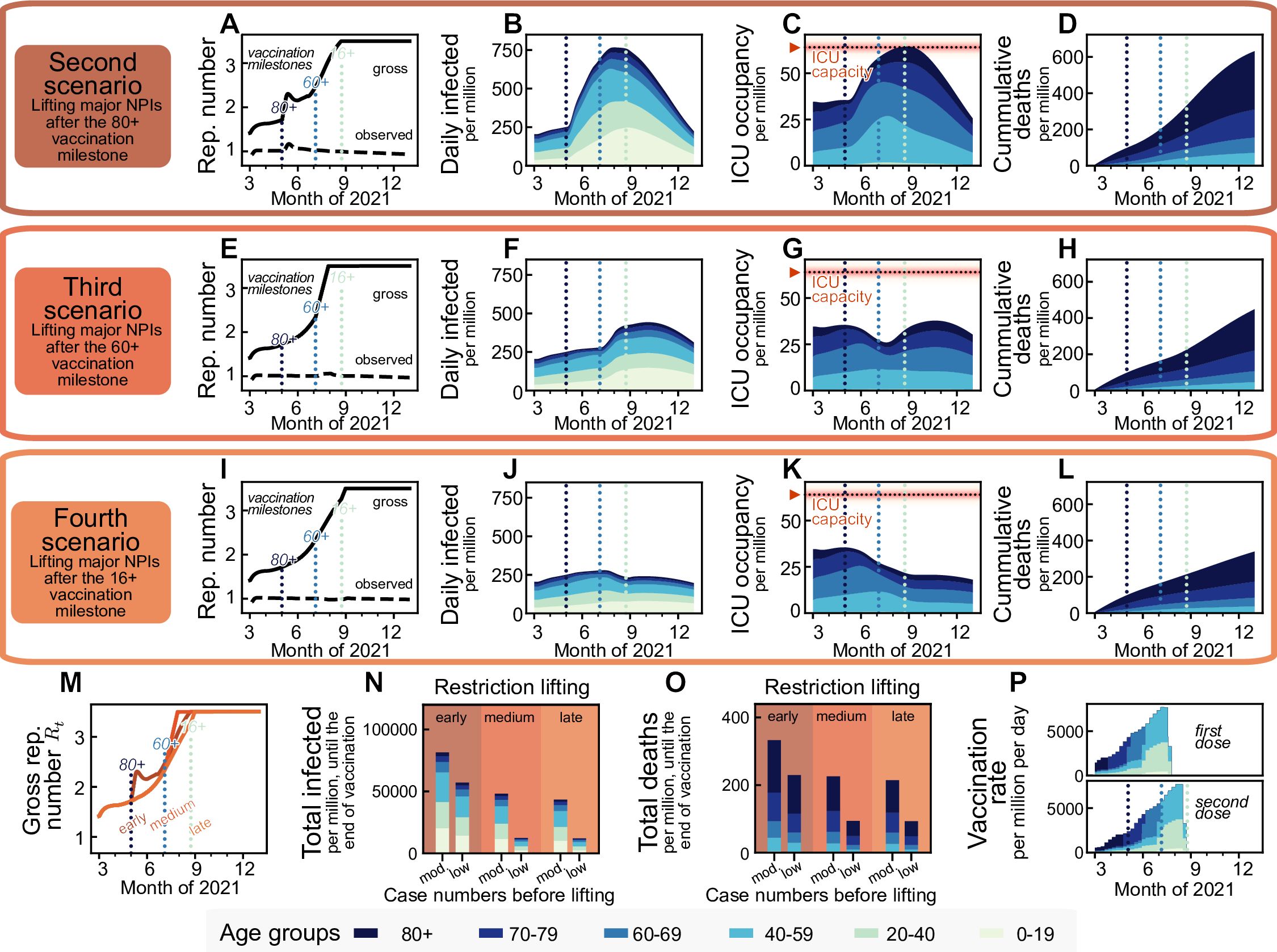

Supplement: S10 Fig — (TIF) [file pcbi.1009288.s010.tif]

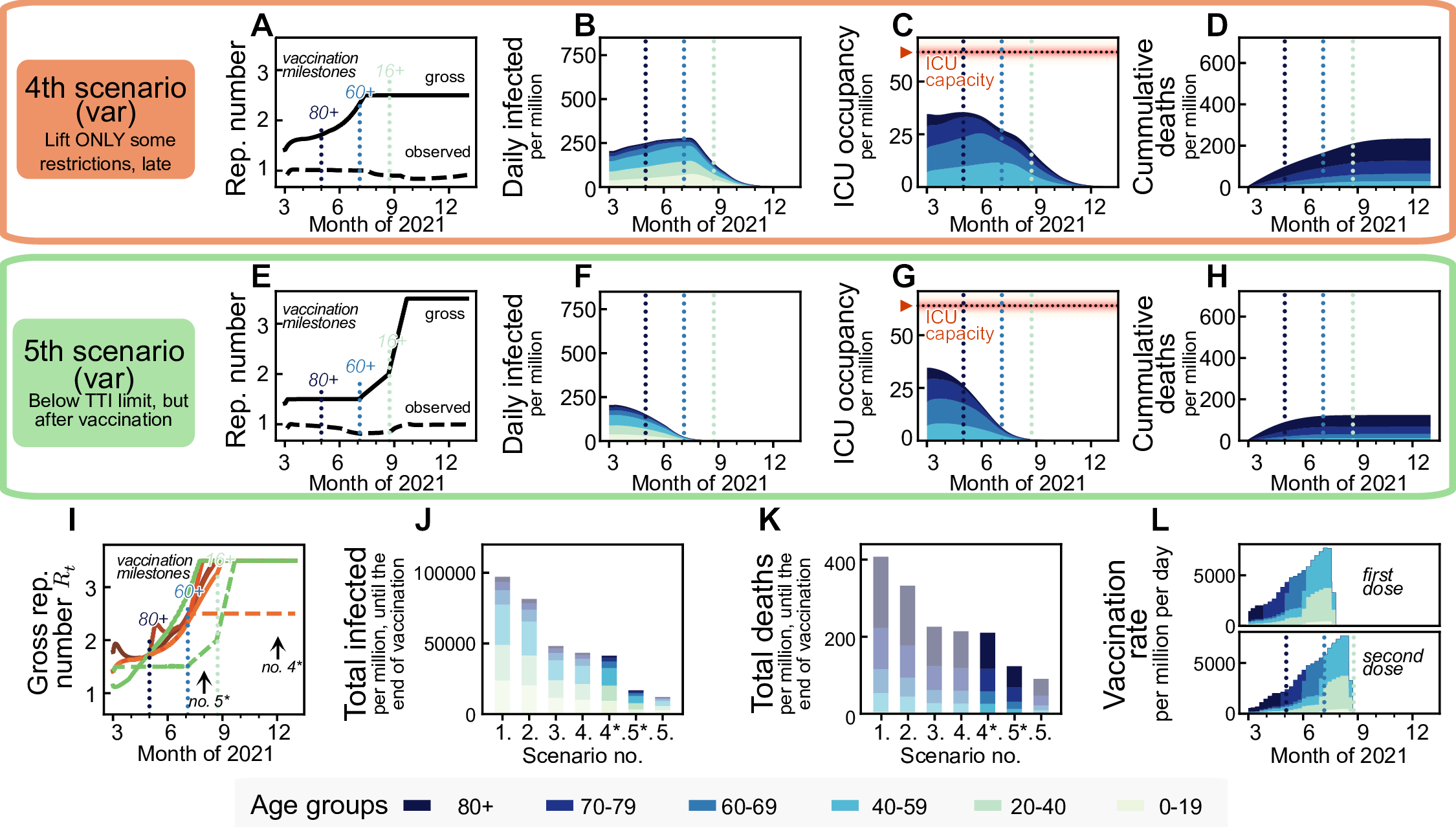

Supplement: S11 Fig — (TIF) [file pcbi.1009288.s011.tif]

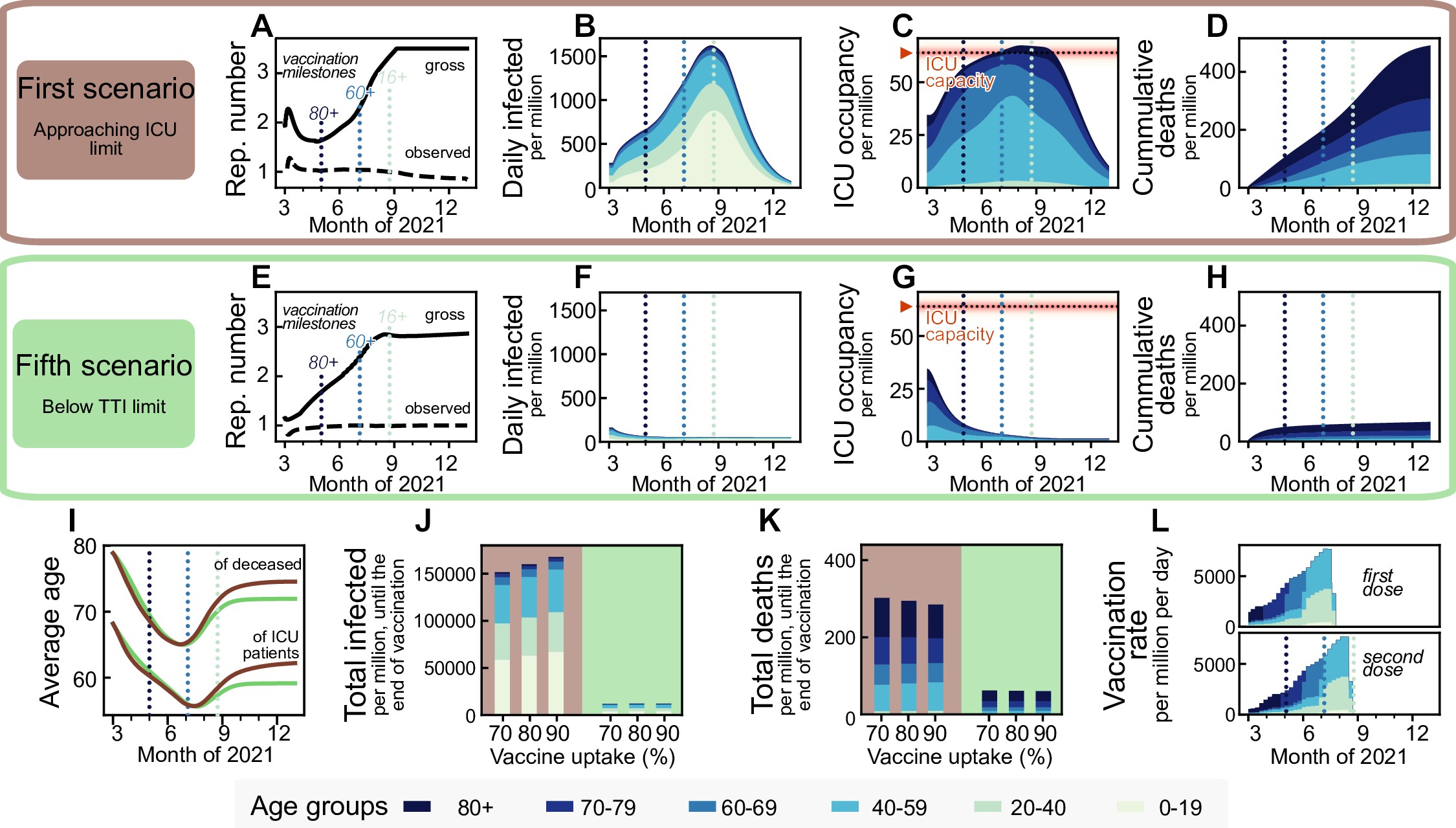

Supplement: S12 Fig — (TIF) [file pcbi.1009288.s012.tif]

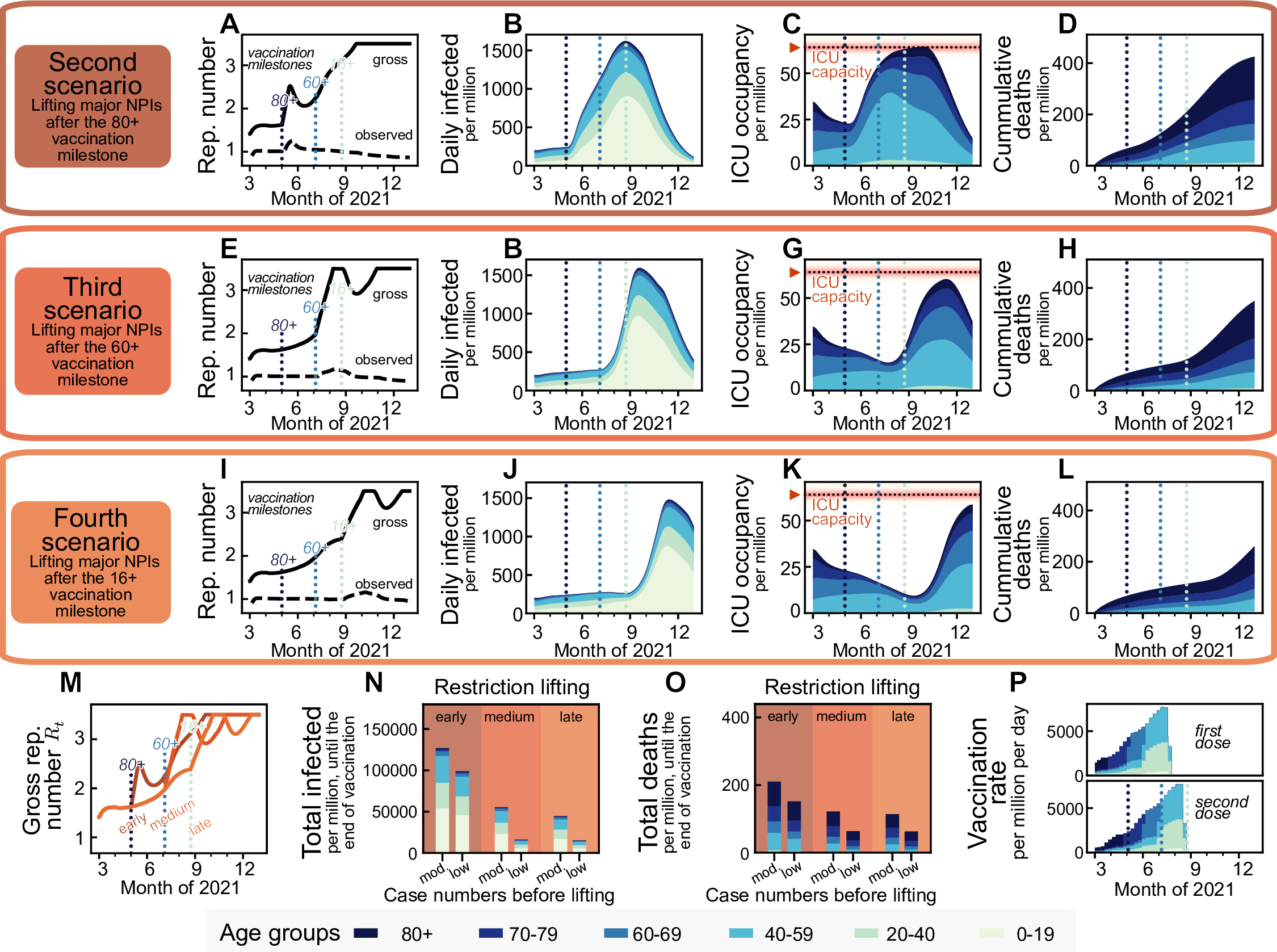

Supplement: S13 Fig — (TIF) [file pcbi.1009288.s013.tif]

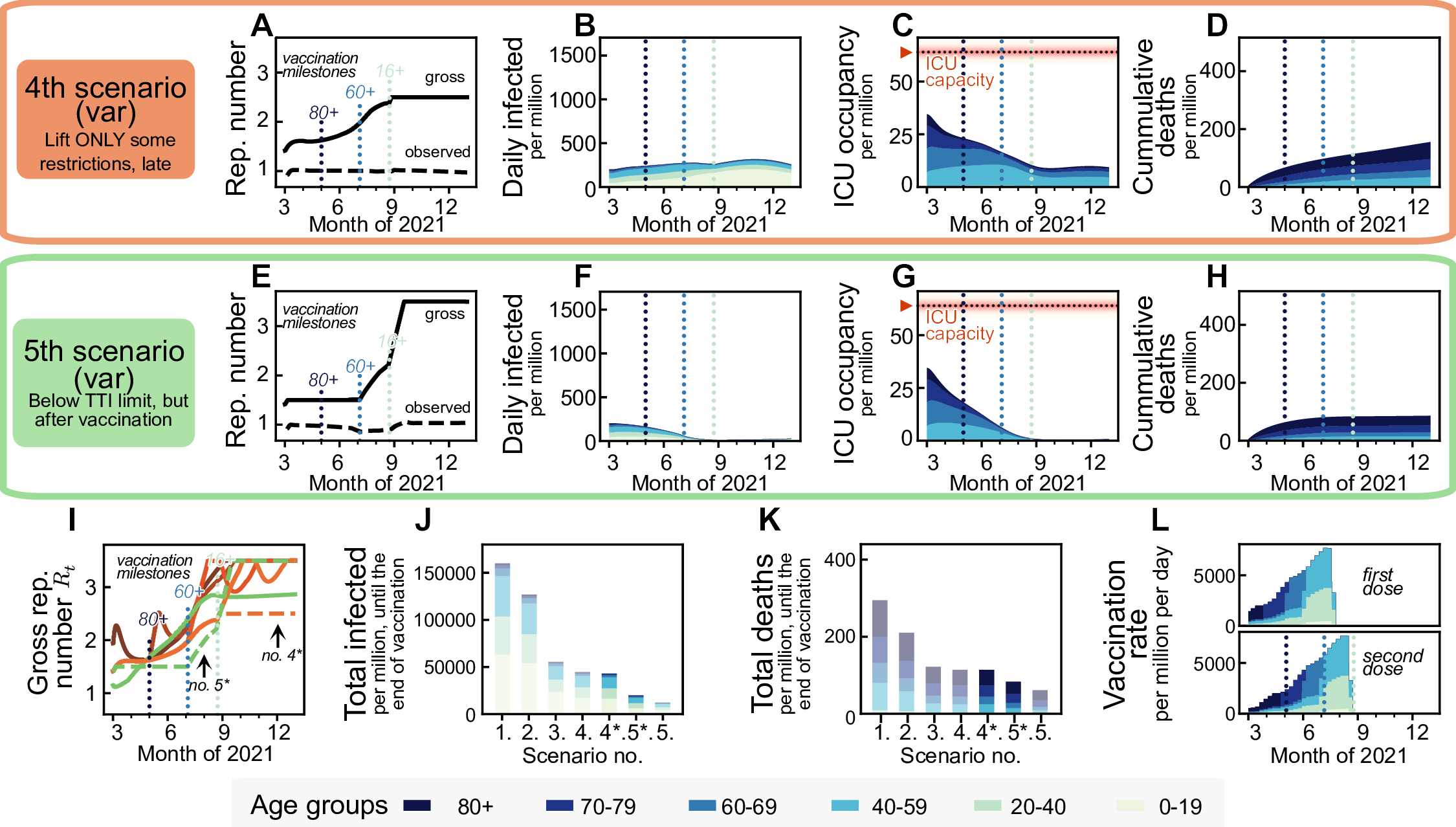

Supplement: S14 Fig — (TIF) [file pcbi.1009288.s014.tif]
