# Supplementary material for: Relaxing restrictions at the pace of vaccination increases freedom and guards against further COVID-19 waves
Source: PLoS Comput Biol. 2021 Sep 2;17(9):e1009288. doi: 10.1371/journal.pcbi.1009288 (PMC8412259; doi:10.1371/journal.pcbi.1009288)
Supplement: S1 Supplementary Note — Here we demonstrate a general case for the eigenvalues of a homogeneous contact matrix, for which every column accounts for the fraction age-groups represent respect to the total population. (PDF) [file pcbi.1009288.s018.pdf]

S1 Eigenvalues of the homogeneous contact matrix

Here we will demonstrate a general case for the eigenvalues of a homogeneous contact matrix, for which every column accounts for the fraction age-groups represent respect to the total population.

Theorem S1.1. *Let C be a square $n \times n$ matrix, such that all columns are identical, i.e., $C_{i,\bullet} = f_i$, $f \in \mathbb{R}^n$, and $\sum_i f_i \neq 0$. Then C is diagonalizable and has a single non-zero eigenvalue $\lambda = \sum_i f_i$.*

Proof. First, we note that the dimension of the kernel of $T : \mathbb{R}^n \to \mathbb{R}^n$, $T(u) = Cu$, i.e, the vector space $\ker(T) = \{u | Cu = 0\}$ is $n-1$. Thus, there are $n-1$ linearly independent vectors associated to the eigenvalue $\lambda = 0$, which algebraic multiplicity has therefore to be equal or larger than $n-1$. Then, we study the nature of the characteristic polynomial:

$$p(\lambda) = \det(C - \lambda I) \tag{1}$$

$$= \begin{vmatrix} f_1 - \lambda & f_1 & \dots & f_1 \\ f_2 & f_2 - \lambda & \dots & f_2 \\ \vdots & \vdots & \ddots & \vdots \\ f_n & f_n & \dots & f_n - \lambda \end{vmatrix} \tag{2}$$

$$\xrightarrow{\text{column } j - \text{column } 1, \forall j>1} = \begin{vmatrix} f_1 - \lambda & \lambda & \lambda & \dots & \lambda \\ f_2 & -\lambda & 0 & \dots & 0 \\ f_3 & 0 & -\lambda & \dots & 0 \\ \vdots & \vdots & \vdots & \ddots & \vdots \\ f_n & 0 & 0 & \dots & -\lambda \end{vmatrix} \tag{3}$$

$$\xrightarrow{\text{row } 1 + \text{row } j, \forall j>1} = \begin{vmatrix} \sum_i f_i - \lambda & 0 & 0 & 0 & 0 \\ f_2 & -\lambda & 0 & \dots & 0 \\ f_3 & 0 & -\lambda & \dots & 0 \\ \vdots & \vdots & \vdots & \ddots & \vdots \\ f_n & 0 & 0 & \dots & -\lambda \end{vmatrix} \tag{4}$$

The highlighted zone in the determinant corresponds to $-\lambda I_{n-1}$, with I_{n-1} the identity matrix in $\mathbb{R}^{n-1 \times n-1}$. Let $\tilde{I}^j_{n-1}$ be I_{n-1}, but with the j'th row replaced by a row of zeros. Using that $|aA| = a^n |A|$ for an $n \times n$ arbitrary matrix, and that $|D| = \prod d_{ii}$ for a diagonal matrix, we calculate $p(\lambda)$ by minor determinants:

$$p(\lambda) = \left(\sum_i f_i - \lambda \right) |-\lambda I_{n-1}| + \sum_{i=2}^{n} (-1)^{i-1} f_i \cancel{\left| \tilde{I}^j_{n-1} \right|} \tag{5}$$

$$= \left(\sum_i f_i - \lambda \right) (-1)^{n-1} \lambda^{n-1}. \tag{6}$$

As we found the last eigenvalue, and, by definition, it has at least one eigenvector, we completed the required set of n eigenvectors and concluded the demonstration. □

Corollary S1.1.1. *When C is a contact matrix as defined in theorem S1.1 and f accounts for the fraction age-groups represent respect to the total population, the largest eigenvalue of matrix C is 1.*

Proof. Direct from theorem S1.1, knowing that $\sum_i f_i = 1$. □
