# Supplementary material for: Relaxing restrictions at the pace of vaccination increases freedom and guards against further COVID-19 waves
Source: PLoS Comput Biol. 2021 Sep 2;17(9):e1009288. doi: 10.1371/journal.pcbi.1009288 (PMC8412259; doi:10.1371/journal.pcbi.1009288)
Supplement: Attachment — Submitted filename: Revision_Letter_v2.pdf [file pcbi.1009288.s019.pdf]

MAX-PLANCK-GESELLSCHAFT

S. Bauer, ... , V. Priesemann

Max Planck Institute for Dynamics and Self-Organization
Am Faßberg 17
D-37077 Göttingen
✉ viola.priesemann@ds.mpg.de

Göttingen, June 22, 2021

Resubmission of our manuscript to *PLOS Computational Biology*

Dear Prof. Struchiner, dear Prof. Pitzer,

we thank you for inviting our submitted manuscript entitled “Relaxing restrictions at the pace of vaccination increases freedom and guards against further COVID-19 waves”, for revision and further consideration of publication in *PLOS Computational Biology*. Find enclosed a point-to-point response to the reviewer’s comments, a manuscript file with changes highlighted by color, and all the required editorial checklists.

Summary of major modifications:

- As suggested by Reviewers 1 and 3, we have reduced the scope of our quantitative analysis to only countries in the European Union (EU), as we agree that vaccination dynamics have been quite different in the UK. We explicitly extended our analysis to other EU countries (Italy, Finland, and the Czech Republic, Figure 5). Accordingly, we adapted the title of the manuscript, removing ”in Europe” from it.
- As suggested by the Editor and Reviewers, we incorporated a real-world contact structure for our age-stratified model. We analyze the influence of different contact matrices on our results (Figure 6).
- As suggested by the Editor and Reviewers 1 and 3, we explored the sensitivity of our results in a broader parameter space (Figure S1).
- As suggested by Reviewer 3, we clarified and streamlined our nomenclature and narrative around the gross reproduction number R_t.
- Based on recent studies that show that vaccinated individuals with a breakthrough infection carry a lower viral load and are less infectious than unvaccinated people [1, 2] we add a parameter describing this effect on vaccinations into our model.
- As requested by the Editorial Office, the PDF file of our manuscript is now generated using the PLOS LaTeX template.

We thank you for your editorial consideration, and for all the extra effort that you have made to speed up the publication process of our article. We are looking forward to your reply,

Viola Priesemann
(on behalf of all authors)

Reviewer 1

The authors have presented an age stratified compartmental model with a suitable structure to simulate COVID dynamics. This has been used to present a compelling argument for maintaining high levels of NPI measures until case numbers are reduced to a low level and vaccination programs are well advanced, which I believe to be well worth publishing.

> We thank the reviewer for their concise summary of our manuscript and are pleased with their positive evaluation.

My most major criticism is the suggestion that the modeling is "European". While many aspects of the model are certainly applicable most European countries (and even a majority of countries globally) there are also many important heterogeneities between European countries which really need to be considered for this to be presented as a European study, including:

> We agree with the reviewer that we were imprecise with the European framing. We accordingly restricted the scope of our quantitative results to countries belonging to the European Union to remove major heterogeneities and extended the analysis to explicitly evaluate the remaining differences across EU countries (see point-to-point answers below). However, we still consider the qualitative results of our study to be generalizable to a broader scope, as the reviewer has also pointed out. We clarified this as part of a new paragraph in the discussion (p14, between lines 351 and 323), where we explain heterogeneities influencing the generalizability of the results:
>
>> Although most examples are presented for countries from the European Union, our results can also be generalized to other countries. Differences across countries come from i) demographics, ii) varying seroprevalence —which originated from large differences in the severity of past waves—, iii) vaccines (types, availability, delivery scheme, and uptake), as well as iv) capacities of the health systems, including hospitals and TTI capabilities. For the EU, we find that during the mass vaccination phase, all these differences have only a minor effect on the pace at which restrictions can be lifted (cf. Fig. S1). However, differences become evident in the long term when most restrictions are lifted by the end of the vaccination campaigns. [...]

Social dynamics and population demographics– something like Prim et als. mixing matrices should ideally be used;

> We thank the reviewer for pointing this out. We have taken concise action in this regard, repeating all our analysis using real-world contact networks for Germany and other European countries. We used the matrices reported in [3] (and variations of such) to study the different scenarios we propose. The final contact matrices for Germany, Italy, Finland, and the Czech Republic are presented in Figure 6. We also contrasted our prior results using a homogeneous contact structure and confirmed that qualitative trends remain unchanged.

Vaccination speed and coverage –- the UK for instance is very advanced in its program, while many Eastern European countries are not;

> We agree with the reviewer. We redirected the quantitative scope to only countries in the European Union. Because of the joint vaccination program across the EU, countries use the roughly the same distribution of vaccine types and the speed of the vaccination program is comparable, at least as long as it is limited by the availability of vaccines and not the distribution and acceptance. We agree that we did not made this distinction clear and adapted the text accordingly throughout the manuscript to refer only the the European Union and not to Europe.

vaccine uptake – again the UK for instance is already pushing 95% coverage in the over 50s, much higher than the central assumptions presented in this study which are based on German projections;

> We agree with the reviewer on that the final uptake will probably differ across countries in the European Union. As vaccine willingness and hesitancy are difficult to predict, we explore our scenarios using vaccine uptake as a free variable when possible (cf. Figure 4 and new Figure 5). When constraining our quantitative analysis to only EU countries, an uptake of 80% among the eligible, 16+ population seems adequate to describe the conservative trends.

vaccine types used – the study here assumes a 75% efficacy against infection, which may be rather high for countries mainly using the Oxford/Az vaccine;

> We agree that the distribution of vaccine types is very relevant to assess a sensible value for the average efficacy against infection. As we now restricted our scope to the European Union with its joint vaccination campaign, however, we argue that the distribution of vaccine types is rather similar across the countries under consideration. We clarified in the text (p25, between lines 629 and 635) our reasoning behind our chosen values of vaccine efficacies:

> For current COVID-19 vaccines, efficacies against severe disease κ ranging from 70–99% [4, 5, 6, 7, 8, 9, 10] and infection blocking potentials η of 60–90% [11, 12, 2, 13] are reported. The roughly uniform distribution of vaccine types in the European Union (see also Figure 8) consists to a larger part of mRNA-type vaccines for which comparatively high values κ of 97–99% [9, 10] and η of 80–90% are reported. We thus chose the rather conservative 90% for κ and 75% for η as our default values.

TTI effects – these form a key part of the results, but track and trace systems have been employed very differently across many countries and the results would really value some fitting to determine actual effects.

> We thank the reviewer for this observation. We have now performed a sensitivity analysis around TTI parameters (Figure S1), and explained the different contributions to their effect throughout the manuscript. We agree that inferring the effectiveness of TTI would lead to very valuable information, however it is not an straightforward task: The reduction of effectiveness of TTI is only gradual with rising case numbers, such that a comparison across time of effective reproduction numbers are confounded with other evolving factors. Comparing across regions or countries is difficult, as the context is vastly different. In brief, it would require to take into account a large number of confounding variables and allow for regional differences, similar to how the effectiveness of interventions has been inferred in these papers: [14, 15]. Even though we do not explicitly fit our parameters from data, we think that the main contribution of our research is largely independent of it and also that the assumptions regarding the effectiveness of TTI are reasonable, which we discuss more in our previous publication [16].

Model parameters for the study have been taken from a variety of sources, predominantly informed by German data, and while estimates seem reasonable ball parks in most cases i believe this would be a much stronger study if Germany was to be concentrated on as a case study with some evidence of model fitting to actual data preferably with parameter ranges to represent confidence intervals. To maintain a European angle, sensitivity could be presented with values indicated for different countries– though I recognise this may be quite a lot of additional work. At the least it would be nice to see some support for TTI parameters as these seem intrinsic to even schematic results.

> We thank the reviewer for this observation. We have now explicitly analyzed different European countries, including new sections and figures in the manuscript to address differences among them in detail (Figures 5, S1, S3). We also performed a sensitivity analysis of our default parameters to “demographics”, i.e.,

how our results would vary when changing age-group sizes and connectivity resembling that of different EU countries (Figure S1).

A few smaller points.

In figure 2 j,k (and similar panels in other figures) totals are quoted for vaccination period given differing vaccine uptake. I found this quite a misleading comparison given that the period itself is dependent on uptake. Could this be presented instead as totals across 2021 (or some similar fixed period)?

> We thank the reviewer for this important observation. We actually used an invariant period, but did not mention it explicitly; "the end of vaccination" refers to the point on time where all willing population in the default scenario (vaccine uptake of 80%) has been offered the vaccine. Now the caption of Figure 2 J,K reads:
>
> > **J,K**: The total number of cases until the end of the vaccination period (of the 80% uptake scenario, i.e., end of August, the rightmost dotted light blue line in sub-panels **A–H**) differ by [...]

I also noticed a few typos: Line 93 "for begin of March"; line 119 "update"; line 620/621 "because by getting".

> We thank the reviewer for pointing this out. We have corrected it.

Reviewer 2

The authors use a well-validated SEIRD-ICU model to explore the dynamics of balancing increased vaccination with increased mobility in a European setting. They find that, with the restriction that ICU limits are not exceeded, a relatively narrow range of scenarios is possible, since too-fast relaxation will quickly result in overwhelmed ICUs. The recommendation is to keep case counts as low as possible to facilitate contact tracing and reduce deaths. Overall, I thought this was a carefully thought out and executed paper, with important findings that quantify just how narrow the range of policy options is: specifically (comparing Fig. 2a and 2e), the highest-infection and lowest-infection scenarios considered are virtually identical except for a 6-week period at the beginning as vaccination is ramping up.

> We thank Dr. Kerr for his positive and encouraging evaluation of our manuscript.

Although the figures are complex, they are extremely well presented and so can still be easily followed. I tried running the scripts to regenerate them, but was not quite sure how to map the two outputs generated onto the figures in the manuscript. Additional documentation may be helpful on this point.

> We thank the Dr. Kerr for the positive feedback on the figures and for pointing at possible improvements in the documentation of the code. We added to the Readme in the Github repository more detailed instructions on how to run the code and elaborated to which figures in the paper the generated figures correspond to. We furthermore extended one of the scripts (*scenarios.sh*). It now generates a new figure with some of the figure panels in the manuscript which were missing in the original scripts, e.g. the bar plots of total deaths and cases. In addition, a new script reproduces the sensitivity analysis figure in Supplementary Information S2.

MINOR COMMENTS

p. 2, line 35: Perhaps add some qualification to the geographical scope of compliance/fatigue – to my knowledge compliance remains high in some settings, such as Australia, Vietnam, and, recently, Bangladesh, which appears to have reversed its enormous spike in cases via a national lockdown.

> We thank Dr. Kerr for this observation. The referenced publication by Petherick et al. states that —even though there are counter examples— there is the general trend of on average increasing pandemic-policy fatigue worldwide. We see that our sentence was imprecise and thus misleading. We the statement now reads (lines 19–20)
>
> > However, the overall compliance with NPIs worldwide has on average decreased due to a "pandemic-policy fatigue" [17]

p. 4, line 90: You mention 4% of the population already vaccinated in the simulations, but what about seroprevalence/cumulative incidence?

> We thank Dr Kerr for pointing this out. We described in detail the initial conditions in the Methods section, that in the original submission was part of the Supplementary Information. In this version, besides keeping the in-detail description in the Methods section, we also added this information to the "Analytical framework", making explicit that we start the simulations with a seroprevalence of 10%. The sentence between lines 109 and 112 (p6) now reads:
>
> > We start our simulations at the beginning of March 2021, with an incidence of 200 daily infections per million, two daily deaths per million, an ICU occupancy of 30 patients per million, a seroprevalence of 10%, and about 4% of the population already vaccinated.

p. 5, Table 1: It's really interesting to see these data – I hadn't realized that ICU-FR has such a strong age trend. One small point of confusion for me: I would have thought ICU-FR = (IFR) / (ICU probability), but this isn't the case, and indeed the IFR for >80 is higher than the ICU probability. While perhaps not the case in resource-limited settings, in a place like Germany I would've expected the number of ICU cases to exceed the number of deaths for all age groups.

> We thank Dr Kerr for pointing this out. Overall, in Germany, less than half of total deaths occur in ICUs. For example, according to the Daily Situation Report of the Robert Koch Institute [18], by March 2, 2021, comparing cumulative total deaths to Table 4, only 31% of total deaths would occur in ICU settings. Furthermore, from discussions that we had with medical professionals, it can be explained that care homes have had some treatment capacity: severe courses with a low probability of survival remained in the care home and were not subjected to the additional stress of a transfer. Additionally, some care-home patients were not transferred to ICU when the COVID-ICU capacity was exhausted locally, which did happen during the 2nd wave in multiple regions in Germany.

p. 6, line 199: typo, "vaccine update" (uptake?)

> Corrected as suggested.

p. 10, Figure 2: In panels j/k, I'm not sure I understand why "total infected" increases with vaccine uptake, or the meaning of the inset charts. Otherwise, this figure is beautifully clear despite its complexity!

We thank Dr. Kerr for his positive feedback on the Figures. Regarding panels J/K, the "total infected" increase with vaccine uptake because a higher vaccine uptake would reduce the fraction of individuals requiring ICU treatment. Therefore, case numbers would be allowed to increase to a higher level so that the available ICUs are occupied.

p. 11, line 139: "complete relaxation of restrictions should wait until everyone has received a vaccination offer" – by "everyone" do you mean adults? And by "ethical", do you mean in terms of equity? One could also ask, I suppose, whether it truly needs to be "everyone" or just above a herd immunity threshold.

We thank Dr. Kerr for pointing to this highly relevant question. We decided to remove the discussed sentence from our manuscript to avoid ethical details that might fall out of its reach.

p. 11, line 151: You may wish to consider the terms "non-susceptible" or "sterilizing immunity" rather than "sterile", which could have unintended negative connotations.

We thank Dr. Kerr for the suggestion. We have adopted the term "sterilizing immunity" throughout the manuscript.

p. 11, line 153: There is increasing evidence of efficacy against variants, but true that much remains unknown. Perhaps soften this statement slightly.

We have rephrased, as suggested. Now the sentence between lines 197 and 200 (p9) reads:

> However, the possibly reduced effectiveness of vaccines against current variants of concern (VOCs), e.g., B.1.351 and P.1 [8, 19, 20], and potential future VOCs render long-term scenarios about the success of vaccination uncertain.

Reviewer 3

In this manuscript the authors simulated different options for COVID-19 vaccination rollout in Germany, from March 2021, combined with the lifting of non-pharmaceutical intervention measures, subject to different constraints relating to the number of cases and healthcare capacity. The lifting of restrictions has been modelled by increasing the allowed number of contacts per person in the population. The authors aimed to identify the rate at which non-pharmaceutical interventions can be lifted, relative to the rate of vaccination. The central findings that the pace of vaccination determines the speed at which other restrictions can be lifted, and that vaccine uptake needs to be high to avoid further waves of infection, are supported by other studies. I found the manuscript interesting and enjoyed reading it, and all of the results and figures were thoughtfully put together. However I have identified some fundamental issues and questions with how the model is posed and described, that I will outline below.

> We thank the reviewer for their concise summary of our manuscript and are pleased with their positive evaluation.

Major comments

1. My main concern is with key terminology that affects the formulation of the transmission model. Throughout, the authors define Rt as the "relative number of contacts" or the "gross reproduction number", which they have at one point described as being equivalent to the average number of infections generated from a single infected individual in a fully-susceptible population (which is the commonly understood definition of the reproduction number R), but then elsewhere refer to Rt as the number of contacts. This translates as though you are considering contacts and average new infections to be equivalent. Examining the differential equations, R_t seems to take the place of what would usually be formulated as the transmission term (conventionally a beta term to describe transmission) which accounts for both contacts and the risk of infection. Assuming that contacts and average new infections are equivalent is confusing and is not appropriate for SARS-CoV-2.

> We thank the reviewer for pointing out this potential for misunderstanding. We adapted our manuscript to call R_t the gross reproduction number now. We call it "gross" to differentiate it from the effective reproduction number typically used in publications. The difference is that we do not include the reduction of the reproduction number occurring because of, e.g., immunity or TTI effects, to reflect the increase of freedom because of the progressing vaccination. In the model overview, the description of this parameter (lines 54–60, p4) now reads:
>
>> A central parameter for our model is the gross reproduction number R_t. It is essentially the time-varying effective reproduction number without considering the effects of immunity nor of TTI. In other words, R_t quantifies the *average number of contacts* that individuals

have that potentially cause a COVID-19 infection in a fully susceptible population, i.e., the *potentially contagious contacts.* Therefore, an increase in R_t implies an increase in contact frequency or in probability of transmission per contact, e.g., due to less mask wearing.

2. I do not agree that these results are broadly generalisable for Europe without considering the individual epidemic history and vaccination strategies of individual countries. The example and parameters in the manuscript are nearly all related to a single country, Germany, and it would not be appropriate to extrapolate these results across Europe (where epidemic trajectories and vaccination delivery do vary to date, although I appreciate there are some similarities in Western Europe), and this should be reflected in the title and text of the manuscript.

We thank the reviewer for this observation; we agree with the limitation that they point out. We have substantially modified our work (from the title to the Supplementary Information) to quantitatively refer only to countries part of the European Union, which roughly follow the same vaccine delivery schedule. We have evaluated the extent of the influence of different age demographics and contact structures across EU countries using those contact matrices reported by Mistry et al. [3] (cf. Figure 7 of the revised manuscript). Furthermore, we analyzed the sensitivity of our analysis to the most important parameters (see Supplementary Figure S1) to confirm the broader generalizability of the qualitative results.

3. The authors note that they did not include a contact network structure in the transmission model, but instead assumed homogeneous contacts across all groups. Capturing a realistic age-based contact structure is critical for many infectious disease models, including for COVID-19, where the age-based distributions of those at highest risk of severe disease outcomes and those being vaccinated are non-uniform, and the interplay with realistic age-based contact patterns would therefore be important to include.

We thank the reviewer for pointing this out. In this revised version, we have studied different real-world contact network structures and derivations thereof. We compare these results with our prior assumption of a homogeneous contact structure, and the results qualitatively remain unchanged. We selected as a default a configuration such that pre-COVID contacts among the adult population remain the same. However, we halve the equivalent contact intensity (either number of contacts or probability of contagion) in school-aged children. We consider this scenario attainable in real-world settings with minor modifications, thus adding more relevance to the message proposed here.

4. External infection term in the equations. I do not understand this part. Wouldn't this typically be modelled as importations of infections, with increasing likelihood of importation as

restrictions are gradually lifted? Looking at your differential equations in the Supplementary Material, susceptibles are being removed due to external contagion – but if those susceptibles are being infected when abroad, surely they should not be modelled as part of the local susceptible pool?

> We thank the reviewer for pointing this out. By 'external infections', we mean individuals from the local susceptible pool who get infected by external sources; that is either because they got infected when abroad or because individuals from abroad infect individuals from the local susceptible pool. We changed the description of this variable in the parameter table to "Infections from external sources". We do not model a change of these external infections because the measures such as travel restrictions, testing and quarantine at arrival, and restrictions in other countries are often not synchronized with the local lifting of restrictions. As we do not have an evidence-based model of how these parameters change, we assume a constant base rate of externally acquired infections. As a result, case numbers do not tend to zero even under strong local restrictions.

5. Throughout the manuscript you mention the strategy of maintaining low case numbers. It is important that you define what you mean by case numbers. Do you mean infections, symptomatic infections, individuals who have tested positive, or symptomatic individuals who have tested positive? These are quite different things – and will differ based on testing strategies.

> We thank the reviewer for this important point. Throughout the manuscript, we have clarified that by *low case numbers* we refer to *a level (of observed daily new cases) where test-trace-and-isolate (TTI) programs efficiently compensate for local spreading events.* As we do not explicitly distinguish between symptomatic and asymptomatic infections, with *observed daily new cases* we refer to the number of individuals that have been tested positive. We are aware that the definition of low case numbers is rather vague, varies from country to country, and depends on several factors, some of which we describe in detail in our previous work [16, 21].

6. The authors make the recommendation that "Throughout summer, only mild contact restrictions will remain necessary." However, I am not convinced that this recommendation is founded by the research presented within this manuscript if numbers of permitted contacts are not explicitly related to non-pharmaceutical interventions. For example – how do specific measures such as school closures, versus mask-wearing, relate to the absolute number of contacts allowed?

> We thank the review for this point. For our argument, we used the results presented in Sharma et al.'s preprint [15] on the effectiveness of NPIs in Europe's second wave, which build on their previous work [14, 22]. They quantify the

effectiveness of interventions by their percentual reduction of the reproduction number, and model the total reduction as the product of the individual interventions. For example, taking a set of relatively mild restrictions like closing nightclubs, mask-wearing, and restricting gatherings to at most 10 people, which have a effectiveness of 12%, 12%, and 5%, respectively, leads to an overall effectiveness of $1 - 0.88 \cdot 0.88 \cdot 0.95 \approx 26\%$. Closing all educational institutions is estimated by Sharma et al. as having an effectiveness of about 8% for Europe's second wave. As inferring the effectiveness of separate NPIs just from epidemiological data would require highly specific methods, as those used in [23, 15, 14, 22], and it is not the main point of our work, we believe it is beyond its scope. Nonetheless, we have added the following passage between lines 76 and 81 (page 4) that reads:

> This number is informed by the estimates of Sharma et al. [15], who estimate the combined effectiveness of mask wearing, limiting gatherings to at most 10 people and closing night clubs to a reduction of about 20–40%, thus leading to a reproduction number between 2.7 and 3.6. We use a conservative estimate, as this is only a exemplary set of restrictions.

7. Table 1. In O'Driscoll et al (Nature) the estimates of IFR by age are tabulated in 5-year age groupings. How did you translate these to the age groupings in column 2?

The IFRs in column 2 are derived from the O'Driscoll 5-year wide IFR strata, by combining those 5-year IFRs proportional to the German population in the corresponding 5-year age-groups taken from [24].

8. Immunity following natural infection. From my understanding, the authors assume no waning of immunity following natural infection. This is important over the timescales being considered, since the authors assume that 10% of the population has naturally-acquired immunity at the beginning of the simulation. A large proportion of that 10% would have been infected in the first wave and their immunity is likely waning (and is likely less protective against new circulating variants). The authors could consider performing a sensitivity analysis with lower levels of immunity assumed at March 2021.

We thank the reviewer for this important point. We assume that neither post-infection immunity, nor the immunization obtained through the different dosages of the vaccine vanish significantly in the considered time frames. As requested, in this version of the manuscript we include a sensitivity analysis, where we study the effect individual parameters have on our results (Figure S1). When analyzing the effect of naturally-acquired immunity (referred in the Figure and throughout the manuscript as seroprevalence), we confirm that it can have a strong effect to vary the initial seroprevelance significantly. However, since the

waves in winter 2020/2021 in Germany and most other EU countries have been much more severe than the first waves in spring 2020, we assume them to have contributed the most to the initial seroprevelance. With antibody levels having shown to last for up to eight months after infection [25], we think the simplification of lasting immunity is both justified and does not strongly affect the results.

9. In addition – did you explore sensitivity of your results to the values chosen for the external contagion term?

> We thank the reviewer for this point. As mentioned in the preceding point, we performed a sensitivity analysis where we explore the effect of different parameters (including the external contagion term) on our results. We summarized this analysis in Figure S1. Because of the considerably different orders of magnitude between community and external contagion in the scenarios explored, Φ has a very low influence in the expected trends for R_t

10. Figure 1a. You include a schematic representation of seasonality. Did you investigate seasonality of SARS-CoV-2 transmission in your study? I did not identify a seasonal forcing term in your equations. I don't believe that we yet have a scientific consensus on the degree to which SARS-CoV-2 is seasonal, and the peaks observed in some countries in the southern hemisphere in summer (e.g. South Africa) would indicate that at least in the initial pandemic phase (before we approach some kind of endemic transmission), transmission could not be described as being strongly driven by seasonality.

> We thank the reviewer for pointing this out. We did not explicitly investigate seasonality, exactly because we also see a lack of scientific consensus regarding the magnitude of such effect. To make it clear that we do not include seasonality in our model and that the intention is purely schematic, we added the title "Schematic overview of factors influencing long-term measures" to Figure 1A, and the following sentence in the caption:
>
> > Note that seasonality is not explicitly modeled in this work.

11. The mechanism of vaccination needs to be explicitly explained. Lines 461-462. "There is some evidence that the vaccines partially prevent transmission of the disease. We therefore remove a fraction of vaccinated people completely from the disease dynamics by moving this fraction to the recovered compartments". This assumes an "all-or-nothing" vaccine where a proportion of vaccinated individuals achieve complete sterile protection and are therefore removed, and that the remaining individuals have the same risk of infection as the unvaccinated population (but with lower risk of ICU and death). This is in contrast to a "leaky" vaccine where in the vaccinated population, there is a reduced risk of infection in line with

observed efficacy against infection, and that individuals who experience a breakthrough infection then have a further reduced risk of severe disease/hospitalisation/ICU/death. In practice the reality is probably somewhere between the "all-or-nothing" and "leaky" extremes, but the dynamics will be different depending on which mechanism is used. I think what you have done is fine here but should be explained in the manuscript using conventional terminology.

> We thank the reviewer for pointing us to the established terminology. We have adapted and clarified the way we include vaccines in our model, updating accordingly in the Methods section. Now lines 406–414 read:
>
> > Our model incorporates the effectiveness against infection following an 'all-or-nothing' scheme, removing a fraction of those vaccinated individuals to the recovered compartments ($V_i^0 \to R_i^1$ and $V_i^1 \to R_i^2$), thus assuming that they would not participate in the spreading dynamics. However, we consider those vaccinated individuals with a breakthrough infection have a lower probability of going to ICU or to die than unvaccinated individuals, i.e., effectiveness against severe disease follows a 'leaky' scheme. Furthermore, we assume those individuals carry a lower viral load and thus are less infectious by a factor of two [1]. All parameters and values are listed in Table 5.

Minor comments

1. Possibly a misunderstanding by me – but why is the denominator throughout the equations $(S_i + R_i)$?

> We thank the reviewer for pointing this out. The denominator $(S_i + R_i)$ appears in every vaccine-related term in the system of differential equations, as we assume that vaccination will not be selective based on serostatus. In that way, individuals that have already recovered from COVID-19 can also access the vaccine. We included this possibility both graphically in Figure 6, and in the Methods section, Model Overview (394–397):
>
> > Our model includes the effect of vaccination, where vaccines are administered with an age-stratified two-dosage delivery scheme. The scheme does not discriminate on serological status, i.e., recovered individuals with natural antibodies may also access the vaccine when offered to them.

2. Abstract. "despite most of the population still being susceptible" – this is a bit vague/imprecise as the level of susceptibility in the population depends on the protectiveness and duration of immunity following prior infection, the effectiveness of vaccines, and the number of people vaccinated.

We agree with the reviewer in the sense that ”still being susceptible” is rather vague, but that is precisely the point we wanted to make. We do not imply that vaccines would completely reduce the susceptibility in the population, but rather increase the pressure for lifting restrictions. We have rephrased ”still being” for ”remaining”.

> However, as vaccination progresses, demands to lift restrictions increase, despite most of the population remaining susceptible.

3. Methods. You should specify that this is a deterministic, delay differential equation model formulated as a system of ordinary differential equations (I had to go to the supplement to understand what type of model you had used).

We thank the reviewer for their suggestion. As we reordered the sections according to the PLOS Computational Biology formatting, we now mention in the manuscript twice that we are using a deterministic approach. In the ”Analytical framework” (Section #2, lines 50–52), it reads:

> Our analytical framework builds on our deterministic, age-stratified, SEIRD-ICU compartmental model, modified to incorporate vaccination through delay differential equations (schematized in Figure 6).

In the Methods section (last section of the paper, lines 364–365), the additional information reads:

> We model the spreading dynamics of SARS-CoV-2 following a SEIRD-ICU deterministic formalism through a system of delay differential equations.

4. Line 71. Basic reproduction number – at what time point?

The basic reproduction number that we chose is under the assumption that the B.1.1.7 variant is dominant. As it is estimated to be about 40 to 90% more transmissible than the original variant, we chose a value of 4.5 [26, 27]. In large part of Europe, the dominance of B.1.1.7 was reached by the end of February, it is therefore the reasonable choice for the time-period considered in our analysis.

5. Line 72. “We further assume that the reproduction number can be decreased to about 3.5 by hygiene measures, face masks, and mild contact distancing.” Can you provide the logic for choosing this value?

We thank the reviewer for pointing this out. In this revised version, we added an explanation for the logic behind that value between lines 76 and 80 (page 4) that reads:

> This number is informed by the estimates of Sharma et al. [15], who estimate the combined effectiveness of mask wearing, limiting gatherings to at most 10 people and closing night clubs to a reduction of about 20–40%, thus leading to a reproduction number between 2.7 and 3.6. We use a conservative estimate, as this is only a exemplary set of restrictions.

6. Figure 1e. What do the shades of blue bars correspond to?

   We thank the Reviewer for pointing this out. The shades of blue represent the different age groups, and now we added the missing legend.

7. Line 90. March of 2021, I assume?

   Indeed, thank you.

8. Line 92. "(assuming a case under-reporting factor of 2)" – do you have a source for this assumption?

   The factor of 2 is the resulting under-reporting factor beginning of March assuming an infection-fatality ratio as published in [28] and that death numbers are not under-reported. We learned that it also in agreement with the estimated under-reporting factor of 1.8 as estimated by the RKI in Germany from seroprevalence studies until November 2020 [29], which was only recently published (on June 9th). We added this information to the text (lines 112-114):

   > This is comparable to German data (assuming a case under-reporting factor of 2, which had been measured during the first wave in Germany[29])[...]

9. S1.9. In the real world, the disease spread can only be observed through testing and tracing. This is not quite true. Disease would be observed through testing, yes, but also through hospitalisation and death records. The full extent of the spread of infection can be observed through testing and tracing.

   We thank the reviewer for their suggestion. We have rephrased the sentence (lines 719–720) as:

   > In real-world settings, the full extent of the disease spread can only be observed through testing and contact tracing.

10. Overall, I thought the figures were very good, however I found several of the figures difficult to interpret because there were too many panels within each figure (E.g. Figures 2 and 3, Figure S6), so the panels individually became quite small.

We thank the reviewer for the positive evaluation of the figures of our manuscript. We have carefully revised all figures in the manuscript and supplementary materials to standardize —and maximize— the sizes of figure titles, labels, ticks, and legends. Content-wise, we believe the current versions maximize the joint goal of condensing information and displaying it in context.

11. Figures S7 to S9 are missing the colour fill legend.

    We thank the reviewer for pointing this out. We have added the missing legends and carefully checked all figures for similar mistakes.

References

[1] Ross J Harris et al. "Impact of vaccination on household transmission of SARS-COV-2 in England". In: *medRxiv* (2021).

[2] Emma Pritchard, Philippa Matthews, Nicole Stoesser, et al. "Impact of vaccination on new SARS-CoV-2 infections in the United Kingdom". In: *Nature Medicine* (2021), pp. 1546–170X.

[3] Dina Mistry et al. "Inferring high-resolution human mixing patterns for disease modeling". In: *Nature communications* 12.1 (2021), pp. 1–12.

[4] Elisabeth Mahase. "Covid-19: Where are we on vaccines and variants?" en. In: *BMJ* 372 (2021), n597. ISSN: 1756-1833. DOI: 10.1136/bmj.n597. URL: https://www.bmj.com/content/372/bmj.n597 (visited on 03/05/2021).

[5] Noa Dagan et al. "BNT162b2 mRNA COVID-19 Vaccine in a Nationwide Mass Vaccination Setting". en. In: *New England Journal of Medicine* (2021). DOI: 10.1056/NEJMoa2101765. URL: https://www.nejm.org/doi/10.1056/NEJMoa2101765 (visited on 03/07/2021).

[6] Jamie L Bernal et al. "Early effectiveness of COVID-19 vaccination with BNT162b2 mRNA vaccine and ChAdOx1 adenovirus vector vaccine on symptomatic disease, hospitalisations and mortality in older adults in England". In: *medrxiv* (2021).

[7] Fernando P. Polack et al. "Safety and Efficacy of the BNT162b2 mRNA COVID-19 Vaccine". In: *New England Journal of Medicine* 383.27 (2020). PMID: 33301246, pp. 2603–2615. DOI: 10.1056/NEJMoa2034577. eprint: https://doi.org/10.1056/NEJMoa2034577. URL: https://doi.org/10.1056/NEJMoa2034577.

[8] Merryn Voysey et al. "Safety and efficacy of the ChAdOx1 nCoV-19 vaccine (AZD1222) against SARS-CoV-2: an interim analysis of four randomised controlled trials in Brazil, South Africa, and the UK". In: *The Lancet* 397.10269 (2021), pp. 99–111.

[9] Israel Ministry of Health. *Effectiveness Data of the COVID-19 Vaccine Collected in Israel until 13.2.2021.* https://www.gov.il/en/departments/news/20022021-01. Last updated: 2021-02-22, Accessed: 2021-03-03.

[10] Eric J Haas et al. "Impact and effectiveness of mRNA BNT162b2 vaccine against SARS-CoV-2 infections and COVID-19 cases, hospitalisations, and deaths following a nationwide vaccination campaign in Israel: an observational study using national surveillance data". In: *The Lancet* 397.10287 (2021), pp. 1819–1829. ISSN: 0140-6736. DOI: https://doi.org/10.1016/S0140-6736(21)00947-8. URL: https://www.sciencedirect.com/science/article/pii/S0140673621009478.

[11] Matan Levine-Tiefenbrun et al. "Decreased SARS-CoV-2 viral load following vaccination". In: *medRxiv* (2021).

[12] Victoria Jane Hall et al. *Effectiveness of BNT162b2 mRNA Vaccine Against Infection and COVID-19 Vaccine Coverage in Healthcare Workers in England, Multicentre Prospective Cohort Study (the SIREN Study).* en. SSRN Scholarly Paper ID 3790399. Rochester, NY: Social Science Research Network, 2021. URL: https://papers.ssrn.com/abstract=3790399 (visited on 03/07/2021).

[13] Mark G Thompson et al. "Interim estimates of vaccine effectiveness of BNT162b2 and mRNA-1273 COVID-19 vaccines in preventing SARS-CoV-2 infection among health care personnel, first responders, and other essential and frontline workers—eight US locations, December 2020–March 2021". In: *Morbidity and Mortality Weekly Report* 70.13 (2021), p. 495.

[14] Jan M. Brauner et al. "Inferring the effectiveness of government interventions against COVID-19". en. In: *Science* (2020). ISSN: 0036-8075, 1095-9203. DOI: 10.1126/science.abd9338. URL: https://science.sciencemag.org/content/early/2020/12/15/science.abd9338 (visited on 01/30/2021).

[15] Mrinank Sharma et al. "Understanding the effectiveness of government interventions in Europe's second wave of COVID-19". en. In: *medRxiv* (Mar. 2021), p. 2021.03.25.21254330. DOI: 10.1101/2021.03.25.21254330. URL: https://www.medrxiv.org/content/10.1101/2021.03.25.21254330v1 (visited on 06/01/2021).

[16] Sebastian Contreras et al. "The challenges of containing SARS-CoV-2 via test-trace-and-isolate". In: *Nature communications* 12.1 (2021), pp. 1–13.

[17] Anna Petherick et al. "A Worldwide Assessment of COVID-19 Pandemic-Policy Fatigue". In: *Available at SSRN 3774252* (2021).

[18] Robert Koch Institute. *Coronavirus disease 2019 (COVID-19): Daily situation report of the Robert Koch Institute (March 02, 2021).* 2020. URL: https://www.rki.de/DE/Content/InfAZ/N/Neuartiges_Coronavirus/Situationsberichte/Maerz_2021/2021-03-02-en.pdf?__blob=publicationFile.

[19] Daniel M. Altmann, Rosemary J. Boyton, and Rupert Beale. "Immunity to SARS-CoV-2 variants of concern". en. In: *Science* 371.6534 (Mar. 2021), pp. 1103–1104. ISSN: 0036-8075, 1095-9203. DOI: 10.1126/science.abg7404. URL: https://science.sciencemag.org/content/371/6534/1103 (visited on 03/17/2021).

[20] Pengfei Wang et al. "Antibody resistance of SARS-CoV-2 variants B. 1.351 and B. 1.1. 7". In: *Nature* (2021), pp. 1–6.

[21] Sebastian Contreras et al. "Low case numbers enable long-term stable pandemic control without lockdowns". In: *medRxiv* (2020).

[22] Seth Flaxman et al. "Estimating the effects of non-pharmaceutical interventions on COVID-19 in Europe". In: *Nature* (2020), pp. 1–8.

[23] Jonas Dehning et al. "Inferring change points in the spread of COVID-19 reveals the effectiveness of interventions". In: *Science* (2020).

[24] DESTATIS. *14. koordinierte Bevölkerungsvorausberechnung für Deutschland (Coordinated Population Projection for Germany).* https://service.destatis.de/bevoelkerungspyramide/#!a=70,80&l=en&g. Bevölkerungspyramide: Altersstruktur Deutschlands von 1950 - 2060. 2019. URL: https://service.destatis.de/bevoelkerungspyramide/#!a=70,80&l=en&g.

[25] Jennifer M. Dan et al. "Immunological memory to SARS-CoV-2 assessed for up to 8 months after infection". In: *Science* 371.6529 (2021). ISSN: 0036-8075. DOI: 10.1126/science.abf4063. eprint: https://science.sciencemag.org/content/371/6529/eabf4063.full.pdf. URL: https://science.sciencemag.org/content/371/6529/eabf4063.

[26] Nicholas G. Davies et al. "Estimated transmissibility and impact of SARS-CoV-2 lineage B.1.1.7 in England". en. In: *Science* (Mar. 2021). ISSN: 0036-8075, 1095-9203. DOI: 10.1126/science.abg3055. URL: https://science.sciencemag.org/content/early/2021/03/03/science.abg3055 (visited on 03/15/2021).

[27] Sam Moore et al. "Vaccination and non-pharmaceutical interventions for COVID-19: a mathematical modelling study". In: *The Lancet Infectious Diseases* (2021). DOI: https://doi.org/10.1016/S1473-3099(21)00143-2.

[28] Megan O'Driscoll et al. "Age-specific mortality and immunity patterns of SARS-CoV-2". en. In: *Nature* 590.7844 (2021), pp. 140–145. ISSN: 1476-4687. DOI: 10.1038/s41586-020-2918-0. URL: https://www.nature.com/articles/s41586-020-2918-0 (visited on 03/07/2021).

[29] RKI. *Corona-Monitoring bundesweit (RKI-SOEP-Studie): Überblick zu ersten Ergebnissen.* https://www.rki.de/DE/Content/Gesundheitsmonitoring/Studien/lid/Ergebnisse.html?nn=14830934. 2021. URL: https://www.rki.de/DE/Content/Gesundheitsmonitoring/Studien/lid/Ergebnisse.html?nn=14830934.
