# Supplementary material for: Relaxing restrictions at the pace of vaccination increases freedom and guards against further COVID-19 waves
Source: PLoS Comput Biol. 2021 Sep 2;17(9):e1009288. doi: 10.1371/journal.pcbi.1009288 (PMC8412259; doi:10.1371/journal.pcbi.1009288)
Supplement: Attachment — Submitted filename: revision_letter.pdf [file pcbi.1009288.s020.pdf]

S. Bauer, … , V. Priesemann

Max Planck Institute for Dynamics and Self-Organization
Am Faßberg 17
D-37077 Göttingen
✉ viola.priesemann@ds.mpg.de

Göttingen, July 15, 2021

Resubmission of our manuscript to *PLOS Computational Biology*

Dear Prof. Struchiner, dear Prof. Pitzer,

we thank you for inviting our submitted manuscript entitled "Relaxing restrictions at the pace of vaccination increases freedom and guards against further COVID-19 waves", for revision and further consideration of publication in *PLOS Computational Biology*. Please find enclosed a point-to-point response to the reviewer's comments and a manuscript file with changes highlighted by color.

In this revised version, we updated the reference list to include those pre-prints that have already been published, and followed all relevant formatting requests for figures and files.

We thank you for your editorial consideration, and for all the extra effort that you have made to speed up the publication process of our article. We are looking forward to your reply,

Viola Priesemann
(on behalf of all authors)

Reviewers 1, 2, 3

> We sincerely thank the reviewers for their positive evaluation of the revisions. We are pleased that they think our manuscript has improved — it is the result of their helpful and constructive feedback.

Reviewer 3

I am satisfied with the responses to the comments and changes made to the manuscript, with the exception of my first major comment. I believe there remains an issue with nomenclature and commonly understood conventions in epidemiological modelling. I apologise if I did not make my comment clear initially.

The reproduction number R_t is described as the "gross reproduction number", i.e. without the impact of immunity, to differentiate it from the effective reproduction number R_{eff}. This is fine. However, in the description, it is now stated that R_t quantifies the "average number of contacts that individuals have that potentially cause a COVID-19 infection in a fully susceptible population, i.e. the potentially contagious contacts". This is incorrect. R_t is widely understood as the average number of new infections generated by a single infectious individual at time t. This is not the same as the average number of contacts that pose a risk of infection – the risk of infection to a susceptible person is driven by transmissibility of the pathogen as well as by the proportion of the population that is infectious (as reflected in your differential equations). Could the authors please correct this definition of the reproduction number?

> We thank the reviewer for their observation. We agree that the way it was described was not the widely understood definition of the reproduction number. As it was presented, R_t could have been interpreted to be proportional to the current incidence. We now define the gross reproduction number R_t as the average number of contacts an infected individual has that would lead to an offspring infection in a fully susceptible population. Besides replacing the use of the phrasing *potentially contagious contacts* throughout the text, we have rephrased the paragraph between lines 54–60 as:
>
>> A central parameter for our model is the gross reproduction number R_t. It is essentially the time-varying effective reproduction number without considering the effects of immunity nor of TTI. That number depends (among several factors) on i) the absolute number of contacts per individual, and ii) the probability of being infected given a contact. In other words, R_t is defined as the average number of contacts an infected individual has that would lead to an offspring infection in a fully susceptible population. Therefore, an increase in R_t implies an increase in contact frequency or the probability of transmission per contact, e.g., due to less mask-wearing.
